# Down-regulation of K_Ca_2.3 channels causes erectile dysfunction in mice

**DOI:** 10.1038/s41598-017-04188-5

**Published:** 2017-06-19

**Authors:** Simon Comerma-Steffensen, Attila Kun, Elise R. Hedegaard, Susie Mogensen, Christian Aalkjaer, Ralf Köhler, Birgitte Mønster Christensen, Ulf Simonsen

**Affiliations:** 1 Department of Biomedicine, Pulmonary and Cardiovascular Pharmacology, Aarhus University, Aarhus, Denmark; 2 Department of Biomedicine, Aarhus University, Aarhus, Denmark; 3 Aragon Agency for Investigation and Development (ARAID), Translational Research Unit, Miguel Servet University Hospital, Zaragoza, Spain

## Abstract

Modulation of endothelial calcium-activated K^+^ channels has been proposed as an approach to restore arterial endothelial cell function in disease. We hypothesized that small-conductance calcium-activated K^+^ channels (K_Ca_2.3 or SK3) contributes to erectile function. The research was performed in transgenic mice with overexpression (K_Ca_2.3^*T/T*(−Dox)^) or down-regulation (K_Ca_2.3^*T/T*(+Dox)^) of the K_Ca_2.3 channels and wild-type C57BL/6-mice (WT). QPCR revealed that K_Ca_2.3 and K_Ca_1.1 channels were the most abundant in mouse corpus cavernosum. K_Ca_2.3 channels were found by immunoreactivity and electron microscopy in the apical-lateral membrane of endothelial cells in the corpus cavernosum. Norepinephrine contraction was enhanced in the corpus cavernosum of K_Ca_2.3^*T/T*(+Dox)^ *versus* K_Ca_2.3^*T/T*(−Dox)^ mice, while acetylcholine relaxation was only reduced at 0.3 µM and relaxations in response to the nitric oxide donor sodium nitroprusside were unaltered. An opener of K_Ca_2 channels, NS309 induced concentration-dependent relaxations of corpus cavernosum. Mean arterial pressure was lower in K_Ca_2.3^*T/T*(−Dox)^ mice compared with WT and K_Ca_2.3^*T/T*(+Dox)^ mice. In anesthetized mice, cavernous nerve stimulation augmented in frequency/voltage dependent manner erectile function being lower in K_Ca_2.3^*T/T*(+Dox)^ mice at low frequencies. Our findings suggest that down-regulation of K_Ca_2.3 channels contributes to erectile dysfunction, and that pharmacological activation of K_Ca_2.3 channels may have the potential to restore erectile function.

## Introduction

Erectile dysfunction (ED) is currently considered as an early clinical manifestation of more generalized cardiovascular disease due to its high prevalence in patients with the major cardiovascular risk factors, diabetes, hypertension, hyperlipidemia, and tobacco abuse^1, 2^. Moreover, ED is an important cause of decreased quality of life in diabetic male patients. In fact, the prevalence of ED is three times higher in men with type 1 and type 2 diabetes than in the general population^3, 4^. Intriguingly, about 50% of the patients exhibit suboptimal responses to oral phosphodiesterase type 5 inhibitors^4, 5^, pointing to a need for alternative treatments that could replace or be adjuvant to current treatments.

Evidence from studies on diabetic patients suggests that endothelial and erectile dysfunction are closely linked to each other^6^. Moreover, endothelium-dependent flow-mediated dilation of the brachial artery was significantly reduced in ED patients^7^ and such impairment of flow-mediated dilatation correlated with the severity of ED^8^. From the pharmacological perspectives, it is therefore worth speculating that an improvement of endothelial function will also improve erectile function.

Impairment of K_Ca_2.3 and K_Ca_3.1 channel activation or gene expression contributes to endothelial dysfunction, and pharmacological activation of these channels has been suggested to improve endothelial function in animal models of cardiovascular disease and diabetes^9, 10^. In rat penile arteries, we found that K_Ca_2.3 and K_Ca_3.1 channels contribute to flow-induced vasodilation and that these dilatations were impaired in type 2 diabetic Zucker diabetic fatty (ZDF) rats^11^. Moreover, in mesenteric arteries from ZDF rats an opener of K_Ca_2.1–3 and K_Ca_3.1 channels restored endothelium-derived hyperpolarization (EDH) type relaxations induced by acetylcholine^12^.

Expression levels of K_Ca_2.3 channels as well as pharmacological activation of the channels may have a strong impact on penile vascular function and thereby erectile function. The major aim of our study was therefore to elucidate the role of K_Ca_2.3 channels in endothelial function in corpus cavernosum and to evaluate whether genetically encoded suppression of K_Ca_2.3 channels contributes to endothelial dysfunction and by this ED. Moreover, we tested whether pharmacological manipulation of K_Ca_2.3 channels could provide a novel endothelium-targeted approach for the treatment of ED.

## Results

### Expression studies

**Figure 1:**
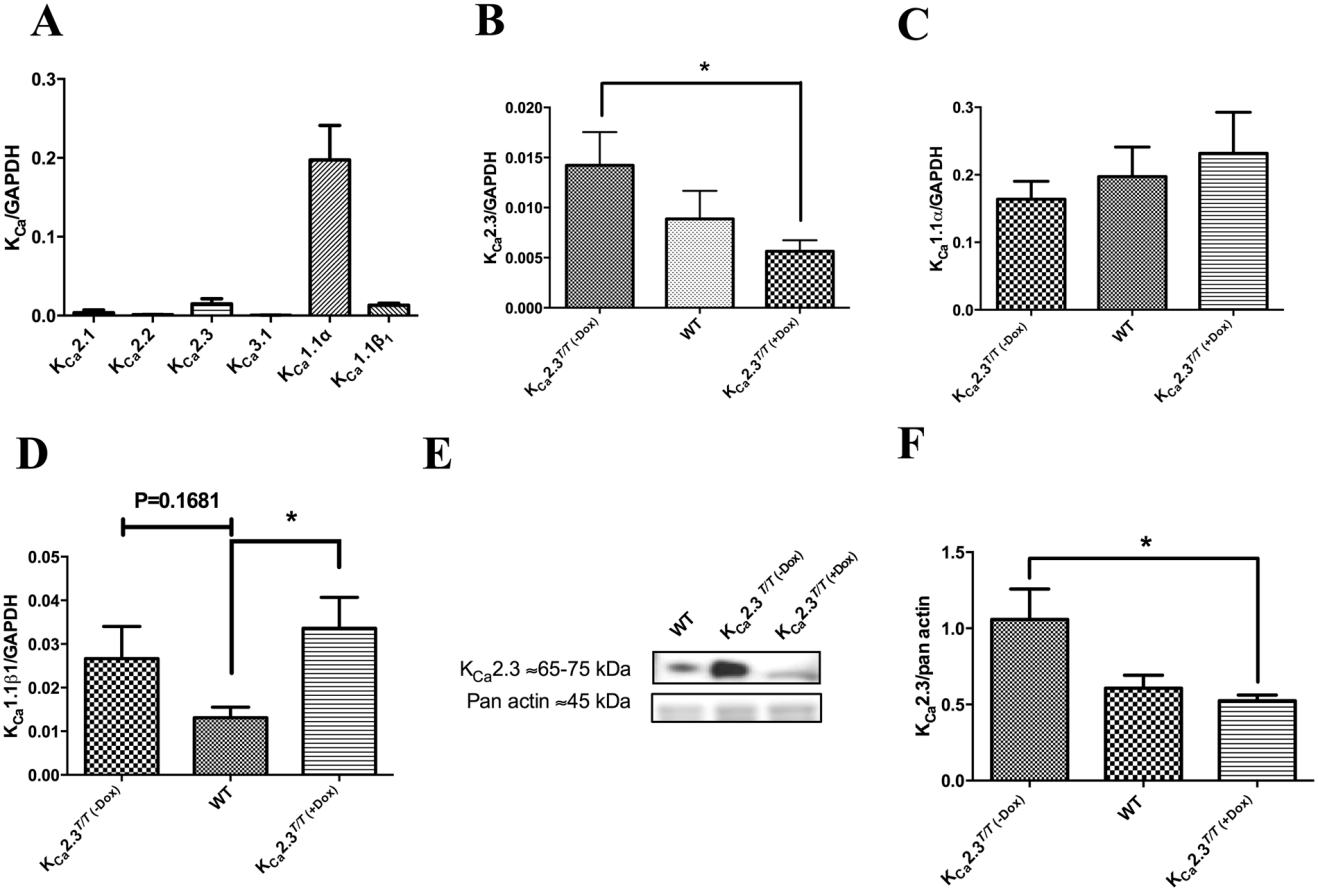
K_Ca_ channel expression in mouse corpus cavernosum. (**A**) Q-PCR showing the expression of K_Ca_ channel subtypes and in case of K_Ca_1.1 subunit α and β1 in corpus cavernosum of wild type mice (WT). Q-PCR showing expression of (**B**) K_Ca_2.3 (**C**) K_Ca_1.1_α_ and (**D**) K_Ca_1.1_β1_ channels in corpus cavernosum from upregulated (K_Ca_2.3^*T/T* (−Dox)^), WT, and down-regulated (K_Ca_2.3^*T/T* (+Dox)^) mice. (**E**) Immunoblot bands for the K_Ca_2.3 channels in corpus cavernosum from K_Ca_2.3^*T/T* (−Dox)^, WT, and K_Ca_2.3^*T/T* (+Dox)^ mice. (**F**) Immunoblot quantification for the K_Ca_2.3 channels in corpus cavernosum from K_Ca_2.3^*T/T* (−Dox)^, WT, and K_Ca_2.3^*T/T* (+Dox)^ mice. Data are means ± SEM of 5–9 animals in each group. P ≤ 0.05 (*) *versus* K_Ca_2.3^*T/T* (+Dox)^ mice. Compared with one-way ANOVA followed by a Tukey multiple comparisons test or with a Student’s t-test. ≈Around.

In corpus cavernosum from WT animals, mRNA expression of the K_Ca_ channels was examined and showed that the K_Ca_1.1_α_ subunit followed by K_Ca_2.3 channels and the K_Ca_1.1_β1_ subunit were the most robustly expressed K_Ca_ channel subtypes (Fig. 1A). Q-PCR showed a clear down-regulation of K_Ca_2.3 mRNA in K_Ca_2.3^*T/T* (+Dox)^ mice (n = 9) as compared to K_Ca_2.3^*T/T* (−Dox)^ mice (n = 9) (Fig. 1B). Expression of K_Ca_1.1_α_ subunits (coding for the pore forming subunit) were unchanged in corpus cavernosum from the three groups of mice (Fig. 1C), while K_Ca_1.1_*β*1_ subunit expression was upregulated in corpus cavernosum of K_Ca_2.3^*T/T* (+Dox)^ versus WT mice (Fig. 1D). Immunoblotting was performed for quantification of K_Ca_2.3 in the corpus cavernosum of K_Ca_2.3^*T/T* (−Dox)^ (n = 5), K_Ca_2.3^*T/T* (+Dox)^ (n = 5), and WT (n = 5) mice and also aorta samples (n = 3–5). The immunoreactive band for K_Ca_2.3 channels in corpus cavernosum from WT mice is slightly heavier than the band for the general K_Ca_2.3^*T/T*^ mice because of the loss of the n-terminal poly-glutamate stretch in these animals (Fig. 1E). We observed a linear relation of pan-actin immunoreaction to the amount of protein loaded (results not shown), and therefore immunoblotting results for K_Ca_2.3 channels were normalized to pan-actin (Fig. 1F) and showed that K_Ca_2.3 expression was lower in corpus cavernosum from the K_Ca_2.3^*T/T* (+Dox)^ mice compared to expression in corpus cavernosum from K_Ca_2.3^*T/T* (−Dox)^ mice (Fig. 1E,F and Supplemental Figure S1E). The same expression pattern was found in aorta samples from WT (n = 5), K_Ca_2.3^*T/T* (−Dox)^ (n = 3), and K_Ca_2.3^*T/T* (+Dox)^ (n = 3) mice (Supplemental Figure S1A and S1E).

Immunoblotting for the pore-forming alpha-subunit of K_Ca_1.1 showed no differences comparing aorta and corpus cavernosum from all three groups of mice (Supplemental Figure S1B and S1C). Immunoreaction for the regulatory beta-subunit of K_Ca_1.1 channels, K_Ca_1.1_β1_, was observed at 28 kDa and 110 kDa. A positive and specific band was supported by previous test using the peptide applied to raise the antibody (Supplemental Figure S1D). The K_Ca_1.1_β1_ showed an expression apparently inverse to up- or down-regulation of K_Ca_2.3 channel expression in corpus cavernosum (Supplemental Figure S1C).

**Figure 2:**
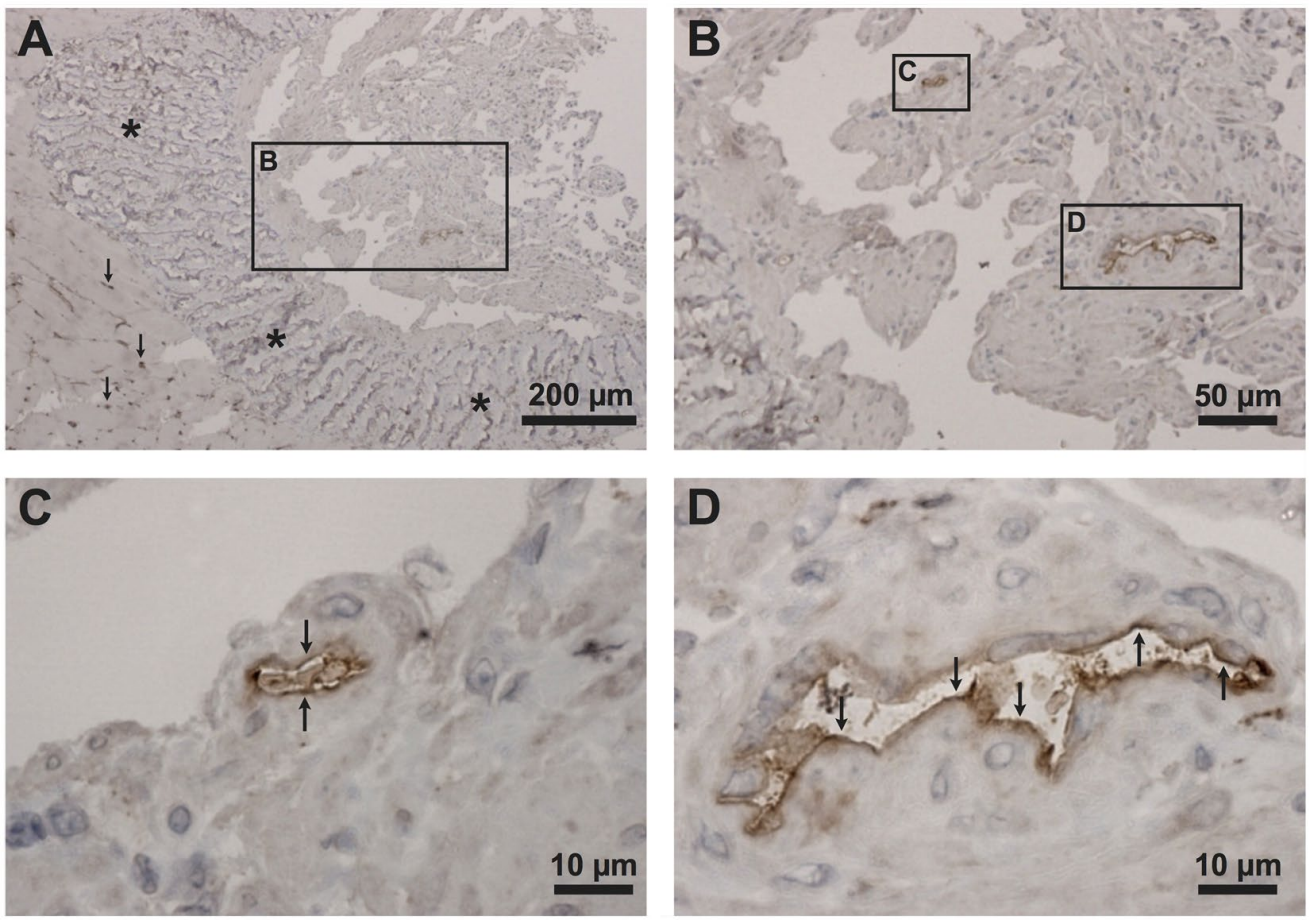
Immunolocalization for K_Ca_2.3 channels in wild type (WT) mouse corpus cavernosum. (**A**) Histological image of penile tissue from a WT mouse. The square area represents part of the corpus cavernosum. The corpus cavernosum is surrounded by the albuginea layer (asterisks) and skeletal muscle. Arrows indicate K_Ca_2.3 labeling in capillaries between the skeletal muscle fibers. (**B**) Histological image showing an enlargement of the square area representing the corpus cavernosum from panel A. K_Ca_2.3 immunoreactivity is seen in blood vessels of the corpus cavernosum (inset **C** and **D**). (**C**) K_Ca_2.3 expression in the apical plasma membrane domains of endothelial cells of a small helicine artery in the corpus cavernosum (arrows, enlargement of inset **C**). (**D**) Similar K_Ca_2.3 expression in the apical plasma membrane domains of endothelial cells of a probable larger artery in corpus cavernosum (arrows, enlargement of inset **D**).

Immunohistochemistry showed expression of K_Ca_2.3 channels in helicine arteries in the corpus cavernosum of WT mice (Fig. 2A,B, panel B shows an enlargement of the inset in panel A). Labelling was also seen in capillaries (arrows, Fig. 2A) between the skeletal muscle fibers surrounding the albuginea layer (asterisks, Fig. 2A) and corpus cavernosum. The K_Ca_2.3 channel was seemly localized in the endothelial cells (arrows, Fig. 2C,D, panel C and D shows enlargements on the insets in panel B).

**Figure 3:**
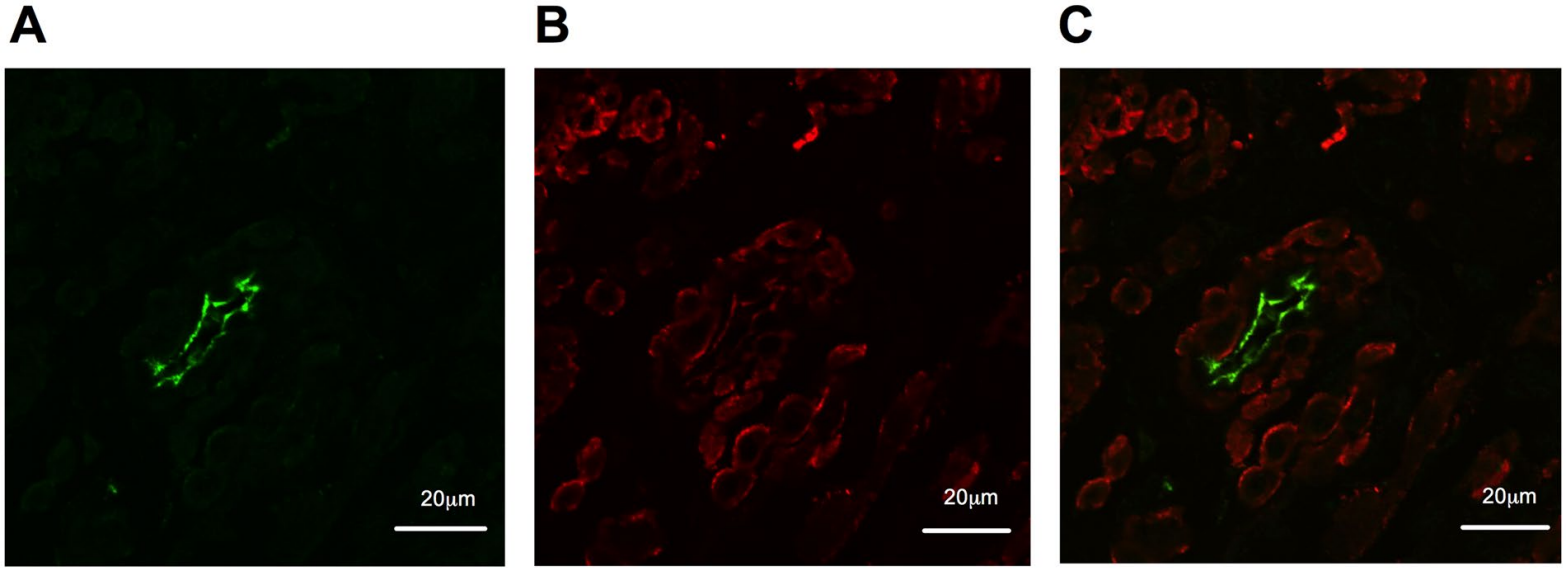
Immunofluorescence staining for K_Ca_2.3 channels in K_Ca_2.3^*T/T* (+Dox)^ mouse corpus cavernosum. (**A**) Immunofluorescence for K_Ca_2.3 expression. (**B**) Immunofluorescence image of smooth muscle actin expression. (**C**) Merged image.

Double labelling for K_Ca_2.3 (green staining, Fig. 3A and C) and smooth muscle actin (red staining, Fig. 3B and C) showed specific K_Ca_2.3 expression in the endothelium of blood vessels and corpus cavernosum (Fig. 3C).

**Figure 4:**
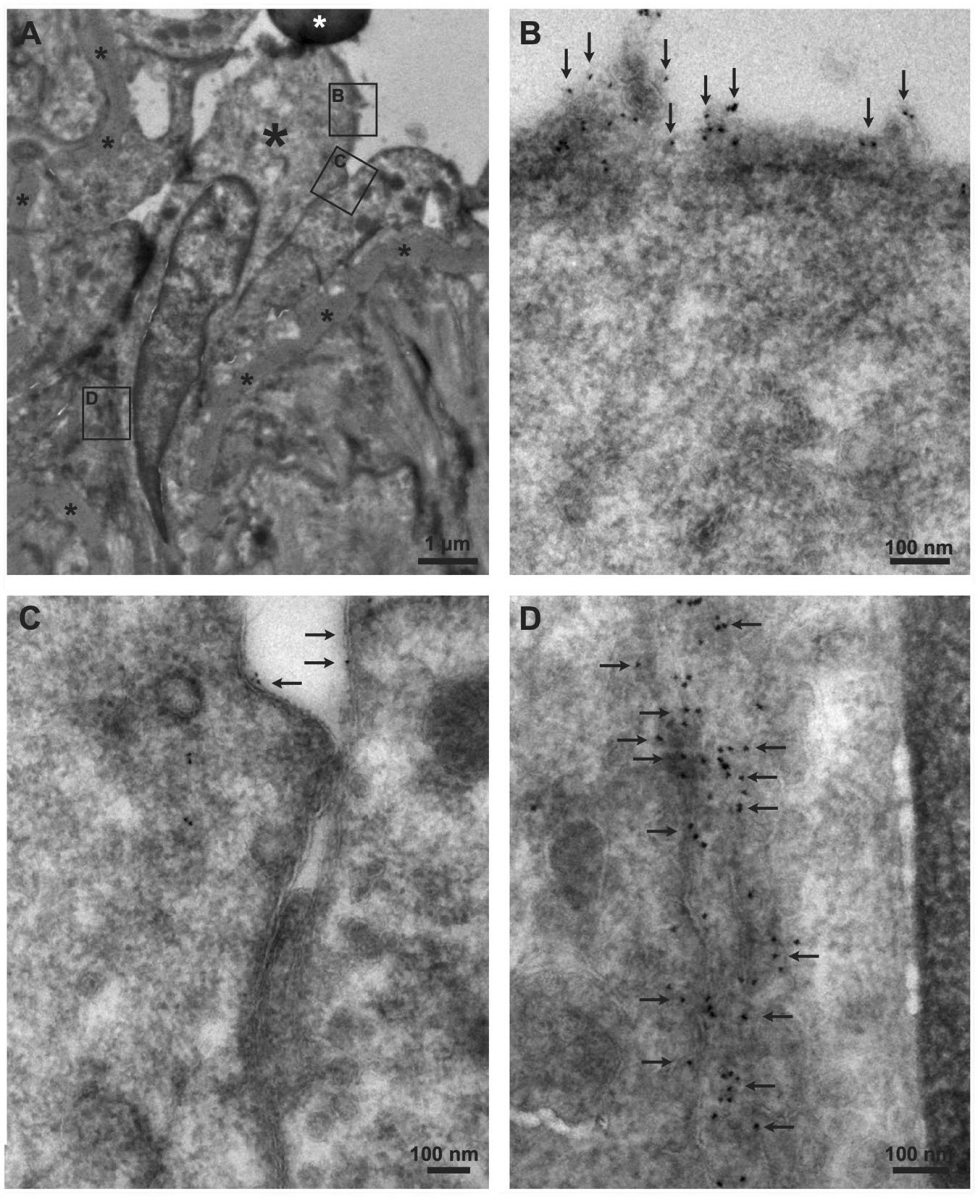
Electron microscopical localization of K_Ca_2.3 in endothelial cells of sinusoids in corpus cavernosum from a wild type mouse. (**A**) Endothelial cells located in a sinusoid of corpus cavernosum (one of the cells is represented by a large black asterisk), small black asterisks represents the basement membrane of the endothelium. White asterisk represents an erythrocyte. (**B**,**C**) K_Ca_2.3 immunoreactivity is seen in the apical plasma membrane of the endothelial cells (represented by 10 nm gold particles, arrows, enlargements of inset **B** and **C**). (**D**) Immunoreactivity for K_Ca_2.3 channels is seen occasionally in the lateral membrane of endothelial cells (arrows, enlargement of inset).

Figure 4 shows immunoelectron microscopical images of endothelial cells lining a sinusoid of the WT corpus cavernosum (Fig. 4A, black asterisk). Gold-particle-labelled K_Ca_2.3 proteins were found on the apical plasma membrane domains of the endothelial cells (Fig. 4B,C). Occasionally, the lateral membrane of the endothelial cells was also labelled with K_Ca_2.3 (Fig. 4D).

### Functional studies in isolated corpus cavernosum strips

**Figure 5:**
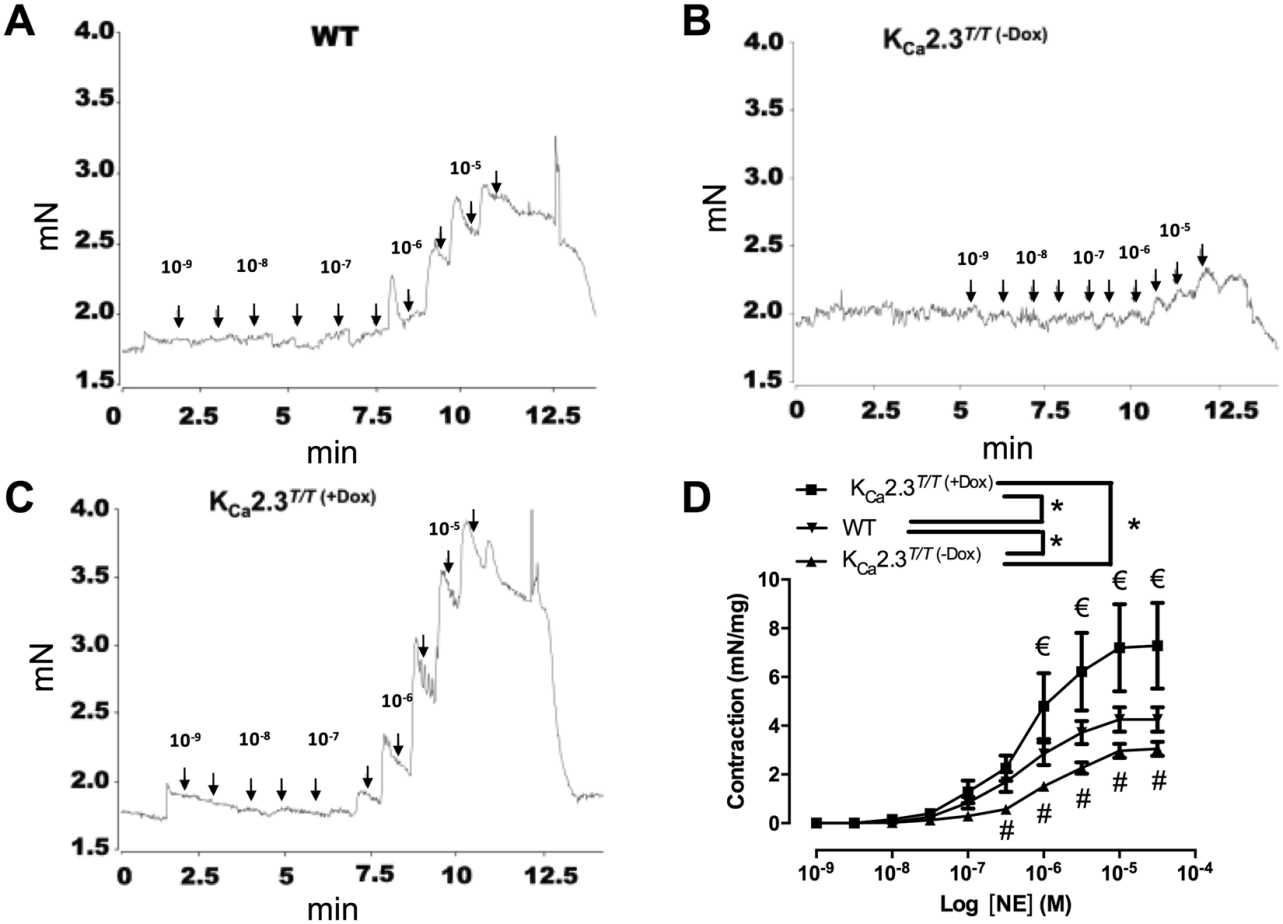
Original traces showing concentration–response curves for norepinephrine (NE) (0.001–30 μM) in corpus cavernosum from (**A**) wild type (WT), (**B**) K_Ca_2.3^*T/T* (−Dox)^ (upregulated model) or (**C**) K_Ca_2.3^*T/T* (+Dox)^ mice (down-regulated model) in mouse corpus cavernosum. (**D**) Average concentration-response curves for NE in corpus cavernosum from (€) K_Ca_2.3^*T/T* (−Dox)^ *vs* K_Ca_2.3^*T/T* (+Dox)^ or (#) K_Ca_2.3^*T/T* (+Dox)^ *vs* WT. Results are means ± S.E.M. P ≤ 0.05 (*), (n = 6–9), two–way ANOVA followed by Tukey *post hoc* test.

**Figure 6:**
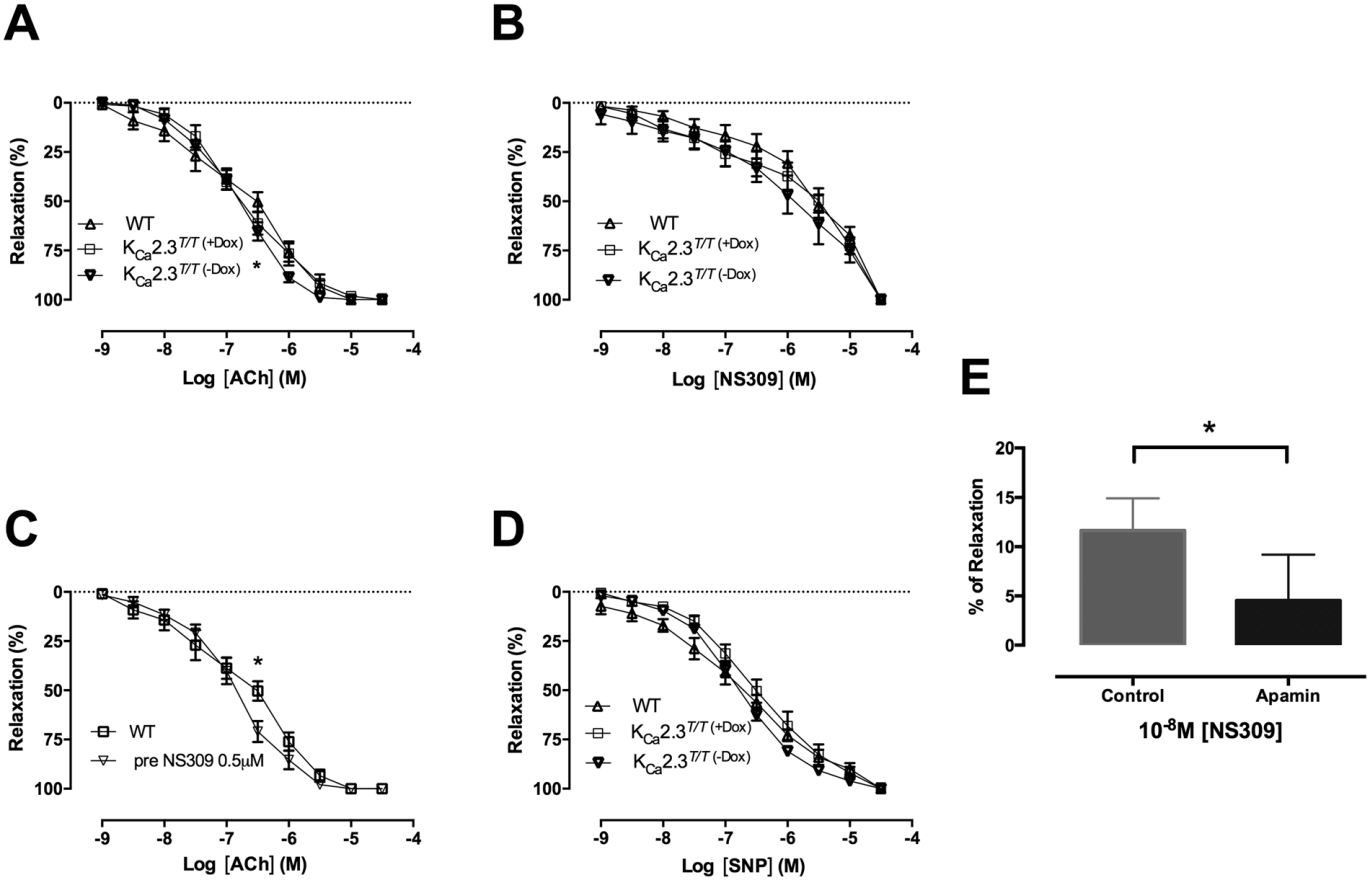
Concentration–response curves for different relaxant compounds (0.001–30 μM) dependent on nitric oxide and K_Ca_2.3 opening in the corpus cavernosum from wild type (WT), K_Ca_2.3^*T/T* (−Dox)^ (overexpressed model) or K_Ca_2.3^*T/T* (+Dox)^ (down-regulated model) in the corpus cavernosum. (**A**) Concentration-response curves for acetylcholine within the K_Ca_2.3 mouse model, (n = 6–9). (**B**) Concentration-response curves for NS309 within the K_Ca_2.3 mouse model, (n = 6–9). (**C**) Concentration-response curves for acetylcholine with and without pre-stimulation with 0.5 μM NS309 in WT mice, (n = 6). (**D**) Concentration-response curves for sodium nitroprusside within the K_Ca_2.3 mouse model. (**E**) Single concentration administration of 0.01 μM NS309 with and without apamin 0.5 μM in WT mice, (n = 6). Results are means ± S.E.M. P ≤ 0.05 (*), (n = 6–9), Student’s t-test or two-way ANOVA followed by Tukey *post hoc* test.

The optimal passive tension for the corpus cavernosum strips examined was similar when comparing preparations from the three groups of mice (Supplemental Figure S2A–F). Therefore, all the experiments were performed with a passive tension of 1.8 mN. Regarding active tension produced by norepinephrine, we found that compared to WT mice, contractions to norepinephrine were enhanced in strips from K_Ca_2.3^*T/T* (+Dox)^ and reduced in strips from K_Ca_2.3^*T/T* (−Dox)^ (Fig. 5). Concentration-response curves for acetylcholine (ACh)-induced nitric oxide (NO)-mediated relaxations were unchanged in corpus cavernosum strips from K_Ca_2.3^*T/T* (−Dox)^, K_Ca_2.3^*T/T* (+Dox)^ and WT mice, but the slopes of the curves were different for K_Ca_2.3^*T/T* (−Dox)^ against the WT, and at 0.3 μM ACh relaxation was significantly enhanced in corpus cavernosum strips from K_Ca_2.3^*T/T* (−Dox)^ mice compared to the WT mice (Fig. 6A).

In corpus cavernosum, NS309, an opener of K_Ca_2 and K_Ca_3.1 channels, induced concentration-dependent relaxations independent of mouse model (Fig. 6B), where apamin significantly inhibited NS309 relaxation at 0.01 µM (Fig. 6E). Incubation with a combination of inhibitors of NO synthase, nitro-L-arginine (L-NOARG, 100 µM) and of cyclooxygenase, indomethacin (10 µM) significantly inhibited NS309 relaxation (Suppl. Figure S3). Although there was no shift in the concentration-response curves for ACh, pre-treatment in WT corpus cavernosum with NS309 in a concentration (0.5 µM) where it is considered selective for K_Ca_3.1 and K_Ca_2 channels^13^, enhanced relaxations induced by 0.3 μM ACh (Fig. 6C).

Concentration-response curves for the NO donor sodium nitroprusside (SNP) were similar in corpus cavernosum from all three groups of mice (Fig. 6D). ODQ, a guanylate cyclase inhibitor, inhibited SNP relaxation to the same degree in corpus cavernosum from all three groups of mice (Supplementary Figure S4A–C).

### *In vivo* erectile function

**Figure 7:**
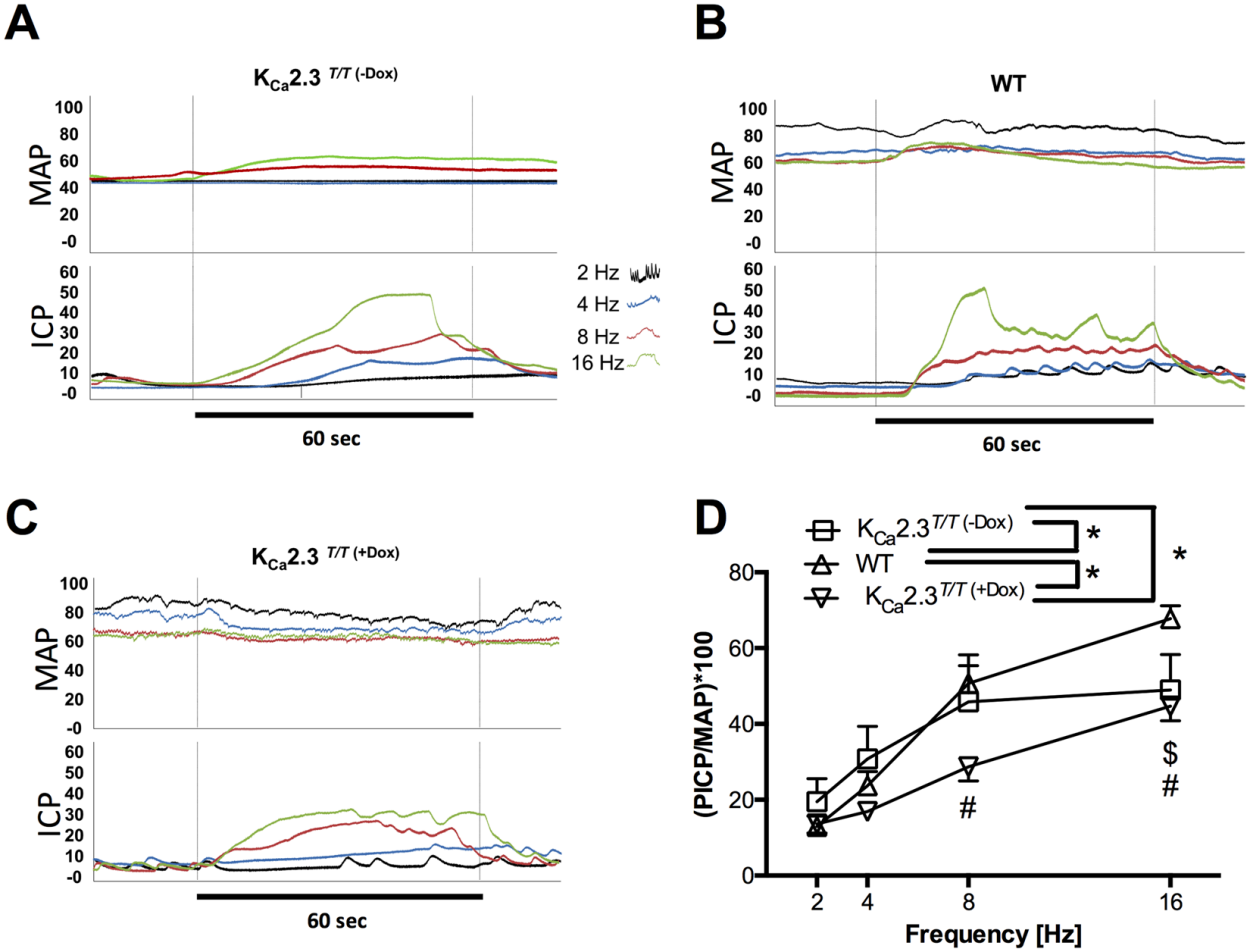
*In vivo* erectile measurements comparing the K_Ca_2.3 mouse models. Mean arterial pressure and intracavernous pressure during cavernous nerve stimulation at 6 V with different frequencies (2, 4, 8, 16 Hz) in (**A**) K_Ca_2.3^*T/T* (−Dox)^ mice with K_Ca_2.3-over-expression. (**B**) wild type (WT) mice. (**C**) K_Ca_2.3^*T/T* (+Dox)^, mice with K_Ca_2.3-down-expression. (**D**) PICP at 6 V with different stimulation frequencies (2, 4, 8, 16 Hz) stimulations from WT *vs* K_Ca_2.3^*T/T* (−Dox)^ ($)/(#) K_Ca_2.3^*T/T* (+Dox)^ mice. Comparisons are made between groups and the same stimulation frequencies. Results are means ± SEM, P ≤ 0.05 (*), (n = 4–8), Two-way ANOVA followed by Tukey *post hoc* test.

Mean arterial pressure (MAP) was decreased in K_Ca_2.3^*T/T* (−Dox)^ mice compared to WT and K_Ca_2.3^*T/T* (+Dox)^ mice (Supplementary Figure S4A). Basal intracavernosal pressure (ICP) was not different among the three groups of animals (Supplementary Figure S5B). Stimulation at 6 V of the cavernous nerve caused frequency-dependent increases in erectile function measured as peak intracavernal pressure (PICP)/MAP. These responses were markedly decreased in K_Ca_2.3^*T/T* (+Dox)^ mice compared to WT, and also compared to the K_Ca_2.3^*T/T* (−Dox)^ mice at 8 Hz (Fig. 7A–C). However, at 16 Hz stimulation, the erectile responses in K_Ca_2.3^*T/T* (+Dox)^ and K_Ca_2.3^*T/T* (−Dox)^ mice were similar and both reduced compared to those in WT mice (Fig. 7D). At lower frequencies there was an enhancement of erectile function in the K_Ca_2.3^*T/T* (−Dox)^ mice (Fig. 7A and D). Stimulations at 1.5 and 3 V showed the same pattern of responses (Results not shown).

## Discussion

The main findings of the present study are that 1) Genetically encoded down-regulation of the K_Ca_2.3 channel in mice results in erectile dysfunction measured as lowered ICP/MAP at 4 and 8 Hz. 2) Electron microscopy revealed that K_Ca_2.3 channels are located primarily on the luminal plasma membrane and occasionally on the lateral plasma membrane of endothelial cells in the corpus cavernosum, and that modulating the expression of these channels (up- or down-regulation) changes norepinephrine contraction in corpus cavernosum strips. 3) A non-selective opener of K_Ca_2 and K_Ca_3.1 channels, NS309, induced concentration-dependent relaxations and enhanced the response to 0.3 µM acetylcholine in corpus cavernosum strips of WT mice. Therefore, suggesting that modulation of these channels may hold the potential for developing a novel approach for treatment of erectile dysfunction.

Previous studies have shown mRNA expression of K_Ca_2.3 channels in rat and human corpus cavernosum^14, 15^. In the present study, Q-PCR showed expression of preferentially the K_Ca_2.3 channel subtype, which suggests that K_Ca_2.3 is the major K_Ca_2 subtype in murine corpus cavernosum.

The K_Ca_2.3 mouse model used in the present study is a conditional model, in which doxycycline treatment causes suppression of the overexpressed channel resulting in expression levels below WT levels^16, 17^. Only few studies had compared against the WT^17, 18^ and biometrical vascular changes are expected on overexpression of K_Ca_2.3 channels^19^. Consequently K_Ca_2.3 currents in endothelial cells were found to be significantly lower than in the WT cells^18^. This is further confirmed by the immunoblotting for K_Ca_2.3 channels in corpus cavernosum from transgenic animals showing upregulation in the vehicle-treated and down-regulation in the doxycycline-treated animals. Certainly, further research in complete K_Ca_2.3 channels deficient models are advised in newer studies.

Immunohistochemical studies have suggested that K_Ca_2.3 and K_Ca_3.1 channels are expressed in the endothelium of rat and human penile arteries^20^. In the present study on murine penile tissue, immunohistochemical staining’s confirmed expression of K_Ca_2.3 channels in endothelial cells of penile arteries and corpus cavernosum. To the contrary, we did not see staining of smooth muscle, suggesting that K_Ca_2.3 channels are mainly expressed in the endothelium of erectile tissue.

K_Ca_2.3 channels have been suggested to be compartmentalized within the endothelial cells of the systemic circulation, and to co-precipitate with caveolin-1, endothelial NO synthase and transient receptor potential channels^21, 22^, suggesting that these proteins interact physically with each other perhaps in caveolae. Other studies have suggested that the K_Ca_2.3 channels are connexin-37 associated and are localized close to endothelial-endothelial cell gap junctions^23^. In the present study, the electron microscopical examinations revealed that in endothelium of corpus cavernosum, there is expression of K_Ca_2.3 channels on the apical or luminal plasma membrane of the endothelial cells and occasionally on lateral membranes of inter-endothelial junctions. In contrast, we found no or only few K_Ca_2.3 channels on basal membranes of the endothelial cells in corpus cavernosum. This localization of the K_Ca_2.3 channels on the endothelial cells in erectile tissue suggests that they may be physically coupled to calcium influx pathways (e.g. calcium-permeable TRPV4 or Piezo-1 channels) on the luminal membrane. On the lateral membrane K_Ca_2.3 channels may be closely coupled to endothelial-endothelial cell communication, but other approaches e.g. measurements of endothelial cell calcium *in situ* and co-staining of channels and myoendothelial gap junctions will be required to clarify this issue.

Suppression of K_Ca_2.3 channels and K_Ca_3.1 channels either by genetic knockdown or their inhibition by the peptide blockers, apamin and charybdotoxin, respectively, have previously been reported to enhance the responses to vasoconstrictors in rat mesenteric arteries^18, 24, 25^, lamb coronary arteries^26^, and neurogenic contractions in rat penile arteries^27^. In the present study, down-regulation of the K_Ca_2.3 channel in mouse corpus cavernosum, markedly enhanced norepinephrine contraction, while the norepinephrine contraction was reduced in corpus cavernosum from mice with up-regulation of K_Ca_2.3. These results suggest that activation and presence of the K_Ca_2.3 channels counterbalances the vasocontraction elicited by the sympathetic neurotransmitter, norepinephrine in corpus cavernosum, that may perhaps provide an important negative feedback on tone and thus favour erectile function.

In systemic arteries, K_Ca_2.3 and K_Ca_3.1 are involved in endothelium-dependent relaxations^18, 28^. The channels also contribute to relaxation in rat penile arteries^27, 29^. Experiments, in which only K_Ca_2 channels were blocked, showed attenuation of acetylcholine relaxations in horse penile arteries as well as in rat corpus cavernosum^30, 31^, suggesting major roles of K_Ca_2.3 channels in relaxation of corpus cavernosum. However, often combined inhibition of both K_Ca_2 and K_Ca_3.1 channels is needed to effectively reduce acetylcholine relaxation^28, 32^, suggesting important roles for both endothelial K_Ca_ channel subtypes in endothelium-dependent relaxation in many vessels. Our present results, provide the first evidence that this may also be true for corpus cavernosum and penile arteries because in the present study down-regulation of K_Ca_2.3 channel expression did not alter relaxation to acetylcholine or to the opener of K_Ca_2/3.1 channels, NS309 in corpus cavernosum from K_Ca_2.3^*T/T* (+Dox)^ mice. Interestingly, we found potentiating effects of NS309 and of upregulation of K_Ca_2.3 channels on ACh-induced relaxation suggesting that K_Ca_2.3 channels could add to acetylcholine relaxation under normal conditions. This further suggests that pharmacological modulation of K_Ca_2.3 channels holds the potential for developing a novel approach for treatment of erectile dysfunction.

In contrast to the effect of apamin on NS309 relaxation, genetic modulation of the K_Ca_2.3 channel failed to cause marked changes in NS309 relaxation. Apart from the K_Ca_2.3 channel we cannot exclude contribution from other apamin-sensitive channels to NS309 relaxation in corpus cavernosum. However, the expression of the K_Ca_2.2 and K_Ca_2.1 channels is markedly lower than of the K_Ca_2.3 channels (Fig. 1A). Therefore, another possibility is that there is an upregulation of the K_Ca_1.1 channel current due to upregulation of the K_Ca_1.1_β1_ subunit. NS309 can lead to release of endothelium-derived NO and prostaglandins followed by activation of smooth muscle K_Ca_1.1 channels^10, 13^. The marked expression of the K_Ca_1.1 channels and our observation that combined inhibition of NO synthase, L-NOARG and cyclooxygenase inhibited NS309 relaxation (Suppl. Fig. S3) support that upregulation of K_Ca_1.1 activity may counteract the effect of downregulation of the K_Ca_2.3 channel on relaxations induced by acetylcholine in corpus cavernosum.

In line with specific roles of K_Ca_2.3 channels in endothelium-dependent vasodilation and norepinephrine-induced tone, K_Ca_2.3 channels have an impact on blood pressure regulation. Indeed, mice with down-regulation of K_Ca_2.3 channel expression have a higher blood pressure^17, 18, 33^. In contrast, up-regulation of K_Ca_2.3 channels has been found to have no effect on systemic blood pressure^18^. This normal blood pressure seems to be caused by higher levels of circulating norepinephrine^34^, which likely counterbalance the tonic vasodilator input provided by K_Ca_2.3 overexpression as observed *in vitro*. Concerning pharmacological experiments, administration of another selective opener of K_Ca_2 and K_Ca_3.1 channels, SKA31, reduces blood pressure over several hours in mice^35^. In conscious dogs, intravenous infusion of SKA-31 produced a strong but short-lived depressor response^36^. In the present study, we also provide evidence that upregulation of K_Ca_2.3 channel expression leads to significantly reduced blood pressure in the systemic circulation, this was evident in the anesthetized mice. Concerning down-regulation of K_Ca_2.3 our study revealed a trend towards systemically elevated pressure, which is in line with previous reports on elevated pressures in the K_Ca_2.3^*T/T* (+Dox)^ mice.

In contrast to the changes observed in the systemic blood pressure, we here found that the basal intracavernosal pressure were similar in the mice with either up- or down-regulation of K_Ca_2.3 channel expression. The basal intracavernosal pressures were low, due to the pronounced activity of the sympathetic nerves to the erectile tissue, when the penis is in the flaccid state^37, 38^. Down-regulation of K_Ca_ channels has also been found to up-regulate sympathetic norepinephrine activity^34^. Although our *in vitro* studies suggest that overexpression of the K_Ca_2.3 channels can inhibit norepinephrine contraction in corpus cavernosum strips, this effect is not reflected *in vivo*, probably due to a low basal pressure in corpus cavernosum *in vivo*.

Drugs currently used to treat erectile function e.g. sildenafil or vardenafil, are phosphodiesterase type 5 inhibitors, which causes relaxation in corpus cavernosum, and penile arteries through increased cyclic GMP also involving activation of K_Ca_1.1 channels^39^. Although the precise mechanism of action needs to be clarified, other drugs, such as calcium dobesilate, can enhance EDH type relaxation in human erectile tissue and restore erectile function in a diabetic rat model by activation of K_Ca_2.3 and K_Ca_3.1 channels^40, 41^. Knockout mice of K_Ca_1.1 channels have reduced erectile function^42^, and openers of K_Ca_1.1 channels can enhance rat erectile function^43, 44^, but so far this is the first study reporting that down-regulation of K_Ca_2.3 channels causes erectile dysfunction in mice.

The expression of K_Ca_2.3 channels in corpus cavernosum from K_Ca_2.3^*T/T* (+Dox)^ mice is downregulated compared to the K_Ca_2.3^*T/T* (−Dox)^ mice, but the downregulation compared to WT mice does not reach significance. In previous studies of K_Ca_2.3^*T/T*^ mice with and without doxycycline-treatment, only few studies have compared the results with wild type mice^17, 18, 45^, and it can be discussed whether the wild type or the upregulated K_Ca_2.3^*T/T* (−Dox)^ mice are the correct controls animals to compare the responses with in animals with downregulation of the K_Ca_2.3 channels. The K_Ca_2.3 ^*T/T*^ channel mice are born with an overexpression of the K_Ca_2.3 channel and that affects probably also the vascular structure and heart during the growth of the animals^45^. At 16 Hz stimulation of the cavernous nerve, the erectile responses are less compared to the WT both in the K_Ca_2.3^*T/T* (−Dox)^ mice and the K_Ca_2.3^*T/T* (+Dox)^ mice. The latter observation may suggest that structural changes influence the erectile response in K_Ca_2.3^*T/T*^ mice compared to control animals. However, compared to the upregulated K_Ca_2.3^*T/T* (−Dox)^ mice, the K_Ca_2.3^*T/T* (+Dox)^ mice have lower erectile responses at lower frequencies of stimulation of the cavernous nerve. Although an instantaneous discharge frequency can reach 35 Hz, the pulse frequency in autonomic nerves rarely exceed 10 Hz^46, 47^, and therefore the findings of lower erectile responses at these frequencies seem relevant. The *in vivo* measurement in the present study were performed in anaesthetized animals and that may also influence the erectile responses, and consequently further investigation in conscious animals using other approaches will be required to confirm that K_Ca_2.3 downregulation and/or pharmacological modulation of K_Ca_2.3 channels play a role for erectile function.

K_Ca_1.1 channels consist of pore forming alpha-subunits and regulatory beta-subunits sensitive to calcium and membrane potential, respectively^48, 49^. Post-transcriptional modulation e.g. sex hormones play a role in regulation of K_Ca_1.1 expression^50^. As far as we understand this is the first time it is reported that a K_Ca_1.1 beta-subunit can be up-regulated by a down-regulated gen and protein expression of K_Ca_2.3 channels. It would be interesting in future studies to examine whether drugs targeting K_Ca_1.1 channels may restore erectile function in mice with down-regulation of K_Ca_2.3 channels.

In contrast to down-regulation of K_Ca_2.3 channels, up-regulation of the channels gave normal intracavernosal pressure responses to low frequency stimulation of the cavernous nerve, while the maximal responses at 16 Hz were reduced compared to the responses in wild-type mice. However, the present study has been performed in healthy animals. Further studies in animal models for cardiovascular disease would be interesting to examine whether erectile function can be restored in diabetes by selective openers of K_Ca_2.3 channels, once they become available.

K_Ca_2.3 channels are also expressed in the brain and in the conduction system of the heart. The effect on the brain can be limited by development of drugs with hydrophilic groups preventing them from crossing the blood brain barrier. In the heart, blockers of K_Ca_2.3 channels have been shown to prevent atrial fibrillation^51, 52^, but currently it is unknown whether specific openers of K_Ca_2.3 channels will *per se* have pro-arrhythmic effects. So far results from experimentation using a non-selective K_Ca_2/3-opener with moderate selectivity for K_Ca_3.1 over K_Ca_2 channels, SKA-31 and SKA-121, in mice and dogs^53^ did not show pro-arrhythmic action of these openers. Regarding K_Ca_2/3 negative gating modulators^54^, a recent study showed that the combined K_Ca_2/3 negative gating modulator, RA-2, has no gross blood pressure elevating effects. However it is worth mentioning that the compound produced mild bradycardia in mice that may reflect a baroreceptor response or prolongation of cardiac action potential duration and thus the cardiac cycle.

In summary, the present study shows that K_Ca_2.3 channels are located in the apical plasma membrane of endothelial cells, and occasionally at inter-endothelial junctions of the corpus cavernosum. We found that down-regulation of these channels increases norepinephrine contraction in corpus cavernosum strips, and it seems associated with erectile dysfunction. Moreover, pharmacological activation of K_Ca_2 channels enhances acetylcholine-induced relaxations in corpus cavernosum, suggesting that modulation of these channels holds the perspectives for developing new drugs and a novel strategy to treat erectile dysfunction.

## Materials and Methods

### Animals and Tissues

Mice were breed at the animal facility of Aarhus University, and animal experiments were performed conform to the guidelines from the Directive 2010/63/EU of the European Parliament on the protection of animals used for scientific purposes. The Animal Experiments Inspectorate from the Ministry of Environment and Food of Denmark approved the study protocol (permissions (2011/561–2011 and 2014-15-2934-01059).

The modified genetically mice used in the present study have a tetracycline-base genetic insertion in a 5′ untranslated region that can be activated by addition of doxycycline (Dox) in the water intake of the animals. For a minimum of 7 days before experimentation Dox (0.5 mg/mL) and sucrose (2%) was administered in dark bottles to the mice. Homozygous K_Ca_2.3 targeted mice (K_Ca_2.3^*T/T*^) with addition (K_Ca_2.3^*T/T* (+Dox)^) or not (K_Ca_2.3^*T/T* (−Dox)^) of Dox, together with their wild-type (WT) littermates were used for experiments. Dox in the water intake was maintained until the research protocols were performed.

For *in vivo* measurements, the mice were anesthetized with intraperitoneal pentobarbital (50 mg/Kg). Pain was assessed regularly during surgery and pressure measurements by pressing a needle against the paw. In case of reaction additional anesthesia (17 mg/Kg pentobarbital) was administered. The mice were cervical-dislocated-euthanized followed by exsanguination after *in vivo* studies or for isolation of tissues and in vitro studies.

Corpus cavernosum was isolated from mice with K_Ca_2.3 overexpression (K_Ca_2.3^*T/T* (−Dox)^, n = 25), down-regulation (K_Ca_2.3^*T/T* (+Dox)^, n = 23), and control wild type mice (WT, n = 24)^16^. Genotyping was performed as previously described^18, 19^. The manuscript followed ARRIVE guidelines^55^.

### Immunoblotting

Corpus cavernosum and aorta from K_Ca_2.3^*T/T* (−Dox)^ mice, K_Ca_2.3^*T/T* (+Dox)^ mice and WT mice were snap frozen and kept at −80 °C in different quantities for K_Ca_ evaluation. Protein was extracted, quantified and mixed in sample buffer before samples were separated by SDS-PAGE and transferred to polyvinylidene fluoride (PVDF) membranes (Bio-Rad). The following protein amounts were used for detection: aorta 12 μg for K_Ca_2.3; corpus cavernosum 12 μg for K_Ca_2.3 (sc-28621), 10 μg for K_Ca_3.1 or 8 μg for K_Ca_1.1_α_ (-alpha-subunit) (Alomone 1184–1200) or K_Ca_1.1_β1_ (-beta-1-subunit) (ab3587); cerebellum and liver 8 μg for K_Ca_1.1_β1_. Samples were incubated with the indicated antibodies, all raised in rabbits: K_Ca_2.3 (1:200), K_Ca_1.1_α_ (1:400), K_Ca_1.1_β1_ (1:500); and housekeeping proteins: pan-actin (1:1000) and beta-tubulin (1:200). Membranes were then incubated with a secondary anti-rabbit IgG (1:4000) and processed with an ECL-Plus kit (General Electric “GE” Health care). Bands were visualized with a luminescence camera (Image Quant LAS 4000 mini from GE) and intensity was quantified by Image Quant TL software (Amersham Biosciences).

### PCR and Q-PCR

Corpus cavernosum tissue was stored in RNA later (Sigma-Aldrich) until extraction and purification of total RNA was performed using the RNeasy Mini Plus Kit (Qiagen). cDNA was synthetized using SuperScript III Reverse Transcriptase (Life Technologies).

The Q-PCR was performed in a MX3005 Q-PCR system (Agilent Technologies). The samples were run for a 40 cycles protocol. Ct-values for the gene of interest were normalised against Ct values for the housekeeping gene (GAPDH), after quantification with the program MxPro v.4.10. (Stratagene, Agilent Technologies). Values are expressed as a ratio of GAPDH. For genotyping of the mice, conventional PCR was performed in a Peqstar thermal cycler (Peqlab). The protocol followed a ‘hot-start’ procedure and thermal cycling conditions.

### Immunohistochemistry

Penile tissue was fixed in 2.5–3% paraformaldehyde overnight and paraffin embedded using standard protocols^56^. Sections were cut, fixed, target retrieve activated and labeled as described in the supplemental protocol for single labeling: primary rabbit anti-K_Ca_2.3 antibody (1:400, Santa Cruz Biotechnology) and secondary goat anti-rabbit peroxidase–conjugated antibody (1:200) were used. Detection was done with 3,3′-diaminobenzidine (DAB) and images were taken using a light microscope (Leica DMRE). For double labeling, sections were incubated with rabbit anti-K_Ca_2.3 antibody (1:400) and mouse anti-smooth muscle actin antibody (1:800, Dako). Visualization was performed with donkey anti-rabbit Alexa Fluor 488-conjugated and donkey anti-mouse Alexa Fluor 555-conjugated secondary antibodies (1:1000, Molecular Probes, Life Technologies). Imaging was obtained with a Leica TCS SL laser scanning confocal microscope and Leica confocal software (Leica).

### Electron microscopy

Penile tissue was maintained in 4% paraformaldehyde in a 0.1 M sodium cacodylate buffer overnight, followed by incubation in 2.3 M Sucrose for 2 hours and snap frozen. Ultrathin cryosections were obtained (Reichert Ultracut S, Leica) and incubated with rabbit anti-K_Ca_2.3 antibody 1:1200) followed by incubation with goat-anti rabbit antibody conjugated to 10 nm gold particles (1:50). The sections were stained for 5 min in a 1.8% methylcellulose/0.4% uranyl acetate solution and observed with an electron microscope (*Morgagni* 268 from FEI Phillips Electron Optics).

### Isometric tension recording in isolated corpus cavernosum

After dissection of corpus cavernosum as previously reported^43, 57^, the strips were mounted between two wire clamps with one clamp connected to an isometric transducer (*Danish Myo Technology*), and immersed in 10 ml of physiological salt solution (PSS), bubbled with a gas mix (95% O_2_ and 5% CO_2_) while kept at 37 °C during the whole experiment^27^.

For strips from WT, K_Ca_2.3^*T/T* (−Dox)^ or K_Ca_2.3^*T/T* (+Dox)^ mice length–contraction curves were constructed with norepinephrine (3 μM) and then acetylcholine ((ACh)−1 μM) to obtain an optimal contraction and relaxation length.

After stable basal tension, corpus cavernosum strips were activated with high potassium salt solution (125 mM KPSS). Afterwards, endothelial function was assessed with norepinephrine (3 μM), and ACh (1 μM). Concentration-response curves (0.001–0.3 μM) were constructed for norepinephrine. The preparations were contracted with norepinephrine to 80% of the maximum response and concentration-response curves constructed for ACh a muscarinic activator, NS309 (6,7-dichloro-1H-indole-2,3-dione 3-oxime) a K_Ca_2 and K_Ca_3.1 opener, and sodium nitroprusside (SNP) a NO donor (0.001–0.3 μM). SNP concentration-response curves were constructed in the absence or in the presence of a guanylate cyclase inhibitor (ODQ 3 × 10^−6^M).

To investigate whether opening of K_Ca_2.1-3 and K_Ca_3.1 channels enhances acetylcholine relaxation, the preparations were incubated with NS309 (5 × 10^−7^ M) prior to construction of concentration-response curves for acetylcholine.

### *In vivo* pressure measurements of intracavernous pressure

Mean arterial blood pressure (MAP) and intracavernous blood pressure (ICP) was measured using catheters placed, respectively, in the carotid artery and corpus cavernosum as previously described^11^. Maximal stimulation (6 V, 1ms, 16 Hz, 60 s) was applied to check maximal erectile function at the beginning of each experiment, before incremental frequencies (2, 4, 8 and 16 Hz) were applied at 1.5, 3, and 6 V. At the end of the experiment the maximal response was repeated to ensure that the cavernous nerve was intact and erectile function maintained.

### Statistical analysis

Statistical comparisons were performed using Graphpad Prism-5.1 (GraphPad Software). Values are presented as means ± S.E.M. QPCR and immunoblotting results were compared with Student’s t-test or in case of three groups with one-way ANOVA followed by Tukey test for multiple comparisons. Norepinephrine-induced-contractions were expressed as mili Newton (mN) of contraction over milligram (mg) of corpus cavernosum dry weight (mN/mg). The responses to ACh, NS309, or SNP were expressed as percentage of relaxation of norepinephrine-(3 μM)-contracted strips. Concentration-response curves were compared using two-way ANOVA followed by a Tukey test or a t-test, when a single concentration between two groups was compared. When the response of a single concentration was examined, with more than two groups, one-way ANOVA followed by Tukey test for multiple comparisons was used. Erectile function was analyzed as the ratio of peak ICP (PICP)(mmHg)/MAP (mmHg) × 100. For each frequency, two-way ANOVA with a Tukey test for multiple comparisons were used. Significance was accepted at P ≤ 0.05.

## Electronic supplementary material

Supplementary Figures and table
