# Supplementary material for: Down-regulation of KCa2.3 channels causes erectile dysfunction in mice
Source: Sci Rep. 2017 Jun 19;7:3839. doi: 10.1038/s41598-017-04188-5 (PMC5476588; doi:10.1038/s41598-017-04188-5)
Supplement: Supplementary file 1 — Supplementary Figures and table [file 41598_2017_4188_MOESM1_ESM.doc]

**Down-regulation of K_Ca_2.3 channels causes erectile dysfunction in mice**

*Simon Comerma-Steffensen^1*^, Attila Kun^1^, Elise R Hedegaard^1^, Susie Mogensen^1^, Christian Aalkjaer*^2^*, Ralf Köhler*^3^*, Birgitte Mønster Christensen*^2^*, and Ulf Simonsen^1^*

^1^Department of Biomedicine, Pulmonary and Cardiovascular Pharmacology, Aarhus University, Denmark; ^2^Department of Biomedicine, Aarhus University; ^3^Aragon Agency for Investigation and Development (ARAID), Translational Research Unit, Miguel Servet University Hospital, Zaragoza, Spain.

**Supplemental Figures and Table**

**
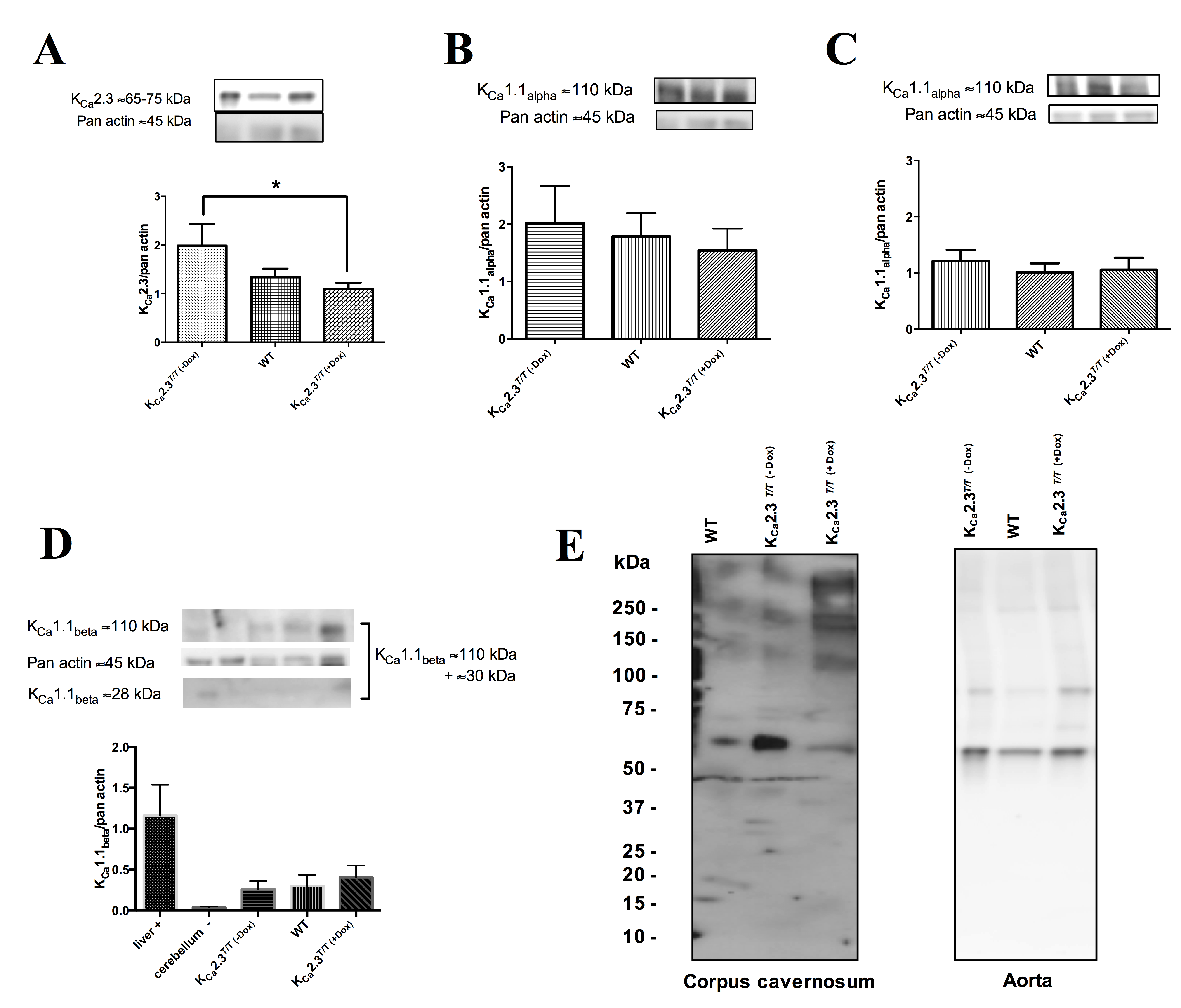
**

**Figure S1.** **Characterization and quantification of K_Ca_ channel expression in the aorta and corpus cavernosum of the mouse.** Western Blot comparison in WT, K_Ca_2.3*^T/T^* ^(-Dox)^, and K_Ca_2.3*^T/T^* ^(+Dox)^ of (**a**) of the K_Ca_2.3 channels in aorta (n= 3–5). (**b**) of the K_Ca_1.1_α_ channels in aorta (n= 6–8). (**c**) of the K_Ca_1.1_α_ channels of in corpus cavernosum (n= 8), and (**d**) of the K_Ca_1.1_β1_ channels in corpus cavernosum (n= 4–5). (**e**) Representative blots from corpus cavernosum and aorta samples in WT, K_Ca_2.3*^T/T^* ^(-Dox)^, and K_Ca_2.3*^T/T^* ^(+Dox)^. Data it is expressed as mean ± S.E.M., P≤0.05 (*). Comparisons are made with one-way ANOVA together with a Tukey multiple comparisons test or with a Student’s t-test. ≈ Around.

**
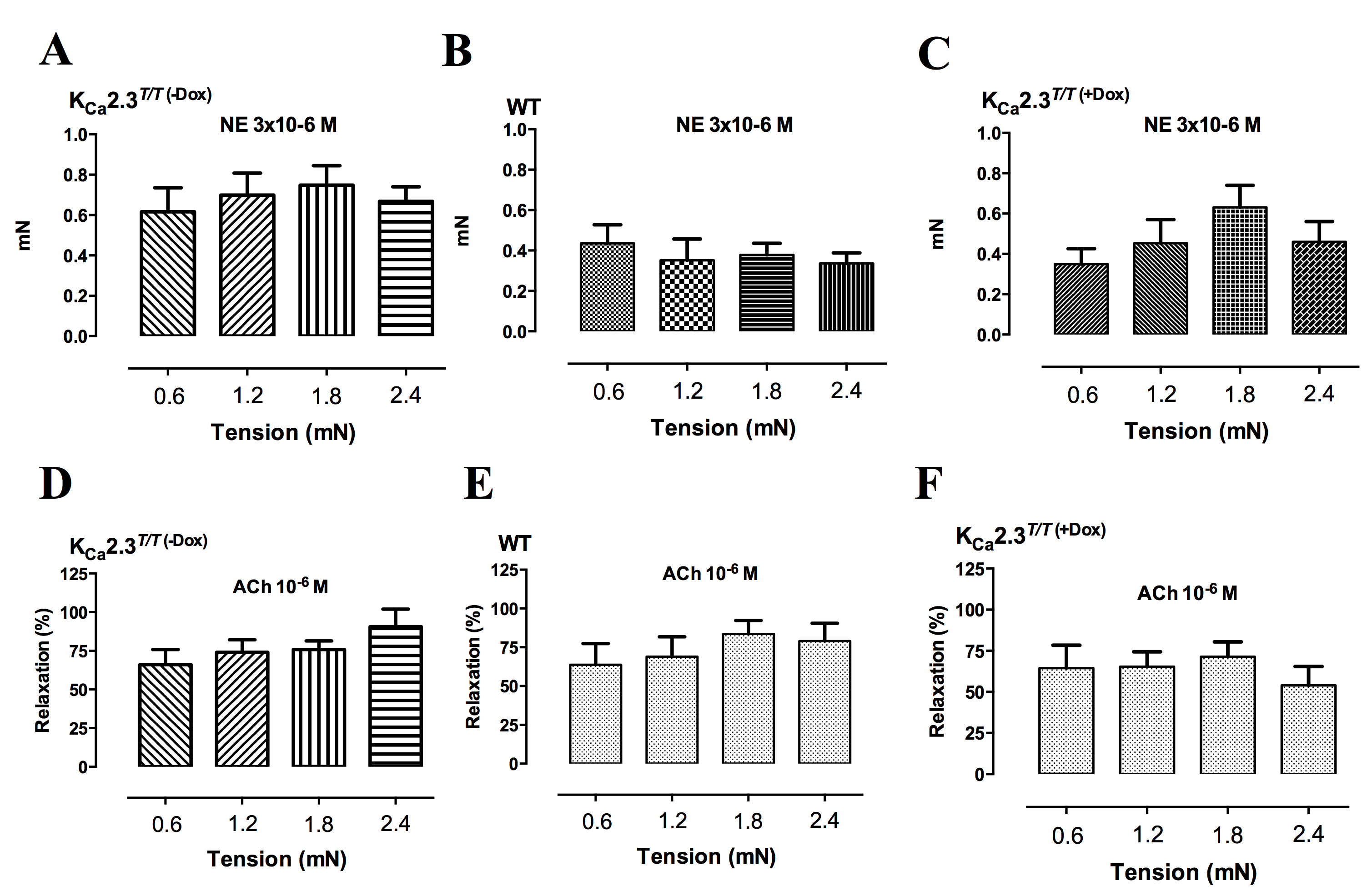
**

**Figure S2. Basal length-tension (0.6–2.4 mN) comparison in corpus cavernosum of K_Ca_2.3*^T/T^* ^(-Dox)^, K_Ca_2.3*^T/T^* ^(+Dox)^ and WT mice.** Norepinephrine (NE) 3x10^-6^ M comparison of length-tension in corpus cavernosum of (**a**) K_Ca_2.3*^T/T^* ^(-Dox)^ mice, (n= 9); (**b**) wild type (WT) mice (n= 8); and (**c**) K_Ca_2.3*^T/T^* ^(+Dox)^ mice, (n= 7). Acetylcholine (ACh) 10^-6^ M comparison between length-tension in corpus cavernosum of (**d**) K_Ca_2.3*^T/T^* ^(-Dox)^ mice (n= 9); (**e**) WT mice, (n= 8); and (**f**) K_Ca_2.3*^T/T^* ^(+Dox)^ mice, (n= 7). Data it is expressed as mean ± S.E.M.


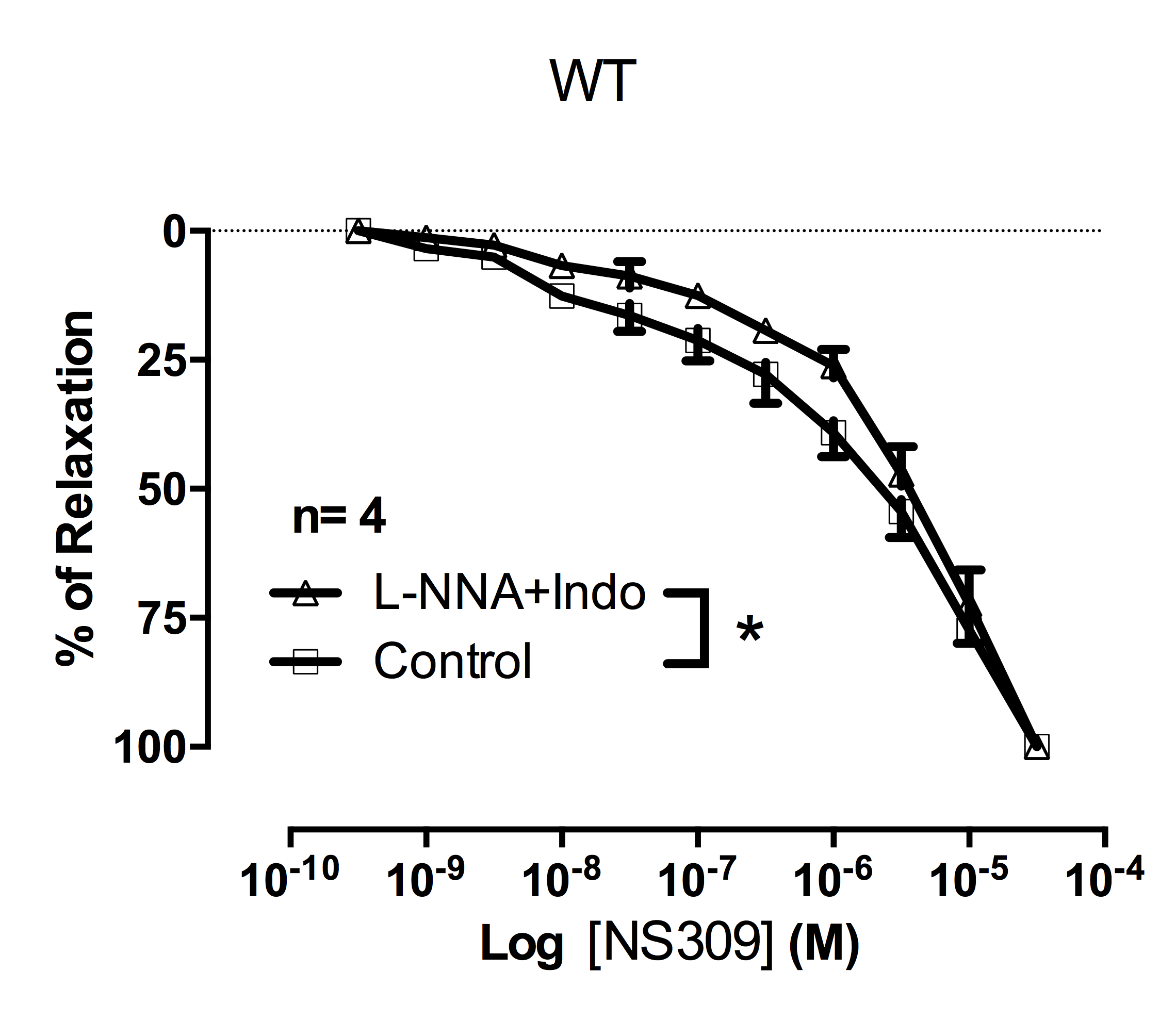


**Fig. S3. Concentration-response curves for NS309 in the absence and the presence of the combination of inhibitors of NO synthase, L-NOARG (100 µM) and of cyclooxygenase, indomethacin (10 µM) in corpus cavernosum from wild type mice.** Results are means ± S.E.M. Significance level P≤0.05 (*), (n=4), two-way ANOVA revealed different concentration-response curves.


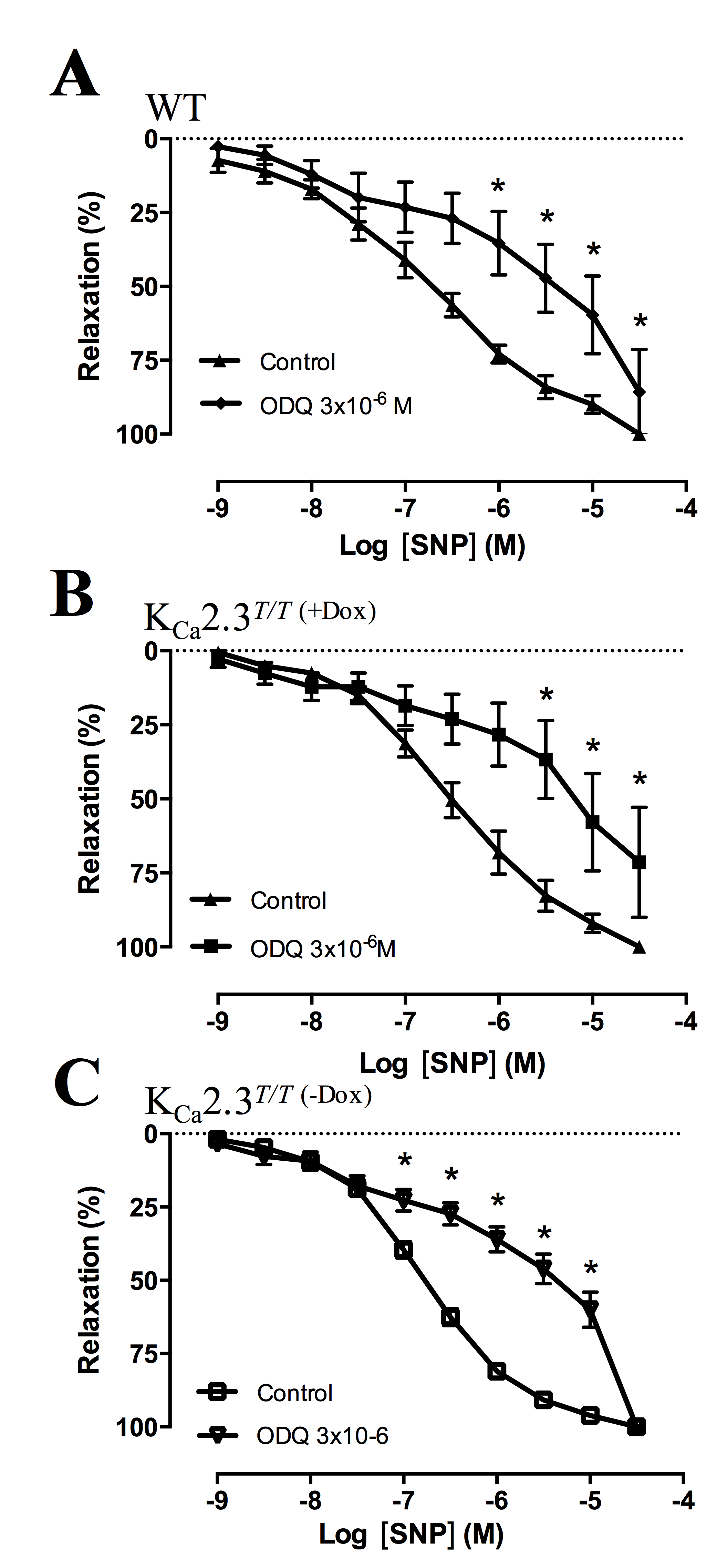


**Figure S4.** **Concentration–response curves for the evaluation of sodium nitroprusside (SNP) (0.001–30 μM) in corpus cavernosum from wild type (WT), K_Ca_2.3*^T/T^* ^(-Dox)^ (overexpressed model) or K_Ca_2.3*^T/T^* ^(+Dox)^ (down-regulated model).** (**a**) Concentration-response curves in corpus cavernosum for sodium nitroprusside with or without ODQ, a blocker of cGMP, in (**a**) WT mice; (**b**) K_Ca_2.3*^T/T^* ^(+Dox)^ mice; (**c**) K_Ca_2.3*^T/T^* ^(-Dox)^ mice. Results are means ± S.E.M. Significance level P≤0.05 (*), (n= 6–9), two-way ANOVA.


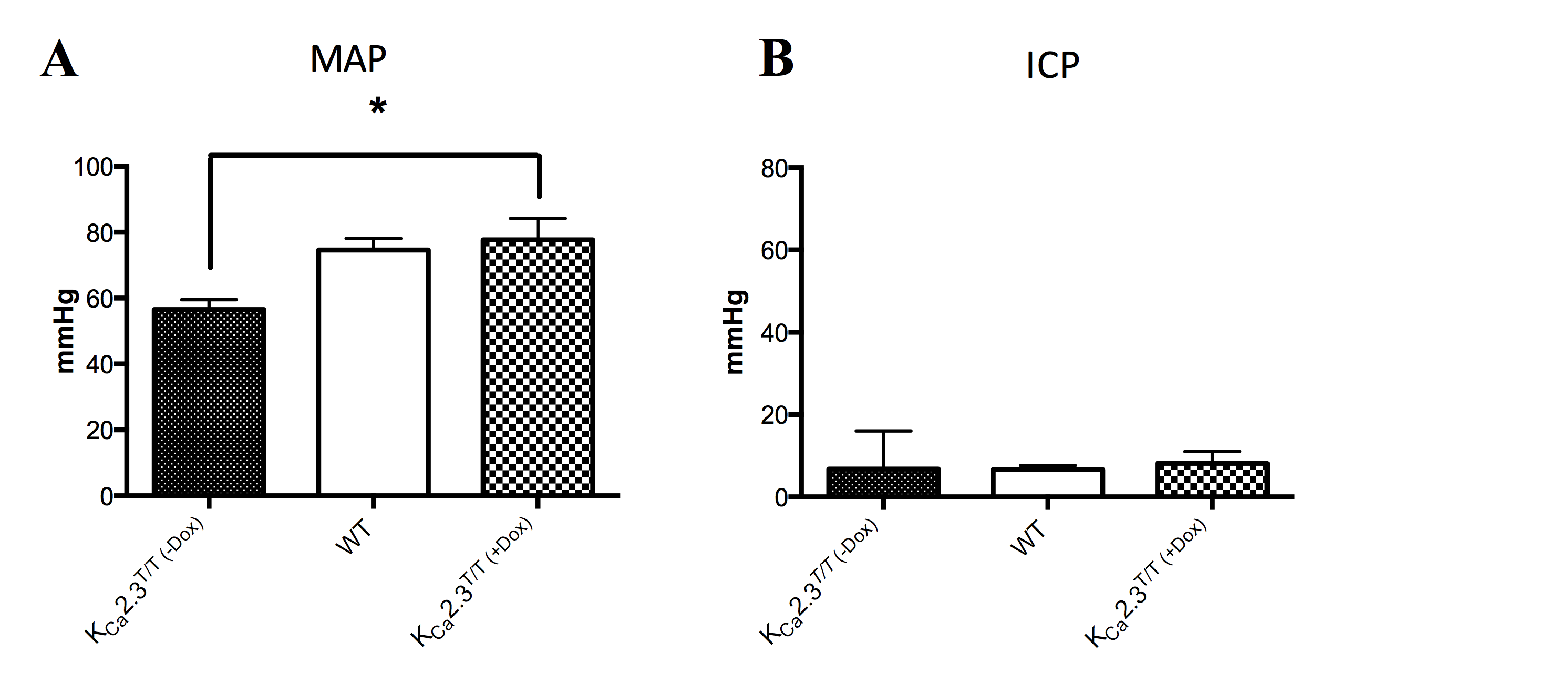


**Figure S5.** ***In vivo* blood pressure parameters in wild type (WT), K_Ca_2.3*^T/T^* ^(-Dox)^ (overexpressed model) or K_Ca_2.3*^T/T^* ^(+Dox)^ (down-regulated model) mice.** (**a**) Mean arterial pressure (MAP) measurements. (**b**) Intracavernous pressure (ICP) measurements. Results are means ± S.E.M. Significance level P≤0.05 (*), (n= 4–8), Student’s t-test.

| Channels | Structure | Identification |
| --- | --- | --- |
| K_Ca_1.1_alpha_ or BK _alpha_ |  |  |
| Forward primer | 5´AGG GGA TGG TGG TTG TTA TGG TG´3 | mKcnma1_3335-F |
| Reverse primer | 5´TGA GGA GTG GGA GGA ATG AGA CAG´3 | mKcnma1_3584-R |
| probe | 5´FAM-TCG TAG GGA GGA TTG GTG ATG-BHQ1´3 | mKcnmaI_3465-P |
| K_Ca_1.1_beta_ or Bk_beta_ |  |  |
| Forward primer | 5´AGG AAG AGC TGG AGG GCA AGA´3 | mKcnmb1_526-F |
| Reverse primer | 5´AGA AGA AGG AGA AGA GGA GGA CTT GG´3 | mKcnmb1_825-R |
| probe | 5´FAM-TTG GCA GAT GTG AAG AAG GTC A-BHQ1´3 | mKcnmb1_687-P |
| K_Ca_2.1 or SK1 |  |  |
| Forward primer | 5´ACC TTC AAC ACA CGC TTC GTC A´3 | mKcnn1_951-F |
| Reverse primer | 5´CAG ACA CAC ACC CTT CCC AC´3 | mKcnn1_1196- R |
| probe | 5´FAM-TCA TCT CCA TCA CCT TCT TGt c-BHQ1´3 | mKcnn1_1119-P |
| K_Ca_2.2 or SK2 |  |  |
| Forward primer | 5´AGG CGT CGC TGT ATT CTT TA´3 | mKcnn2_470-F |
| Reverse primer | 5´AGG TAT GGG ATG GAT AGC AC´3 | mKcnn2_672-R |
| probe | 5´FAM-ACC ACG CCA GGG AAA TAC AGT-BHQ1´3 | mKcnn2_548-P |
| K_Ca_2.3 or SK3 |  |  |
| Forward primer | 5´CAG CTC TTC AGT CCC AGC AA´3 | mKcnn3_933-F |
| Reverse primer | 5´CCA AAC ATC CCA AAA ATC AGA GCA´3 | mKcnn3_1189-R |
| probe | 5´FAM-ACC CCA ACG CTA CCC ACA ACC A-BHQ1´3 | mKcnn3_1015-P |
| K_Ca_3.1 or IK |  |  |
| Forward primer | 5´GCA CAC TCG AAG GAA GGA C´3 | mKcnn4_1149-F |
| Reverse primer | 5´GGT TCT GGT AGC TGC TGT TG´3 | mKcnn4_1431-R |
| probe | 5´FAM-AAT CGA CGG TCT GGC AGG AAa g-BHQ1´3 | mKcnn4_1353-P |

**Table S1**. Sequences for the forward and reverse primers and probes used for K_Ca_ channels PCR experiments in corpus cavernosum from K_Ca_2.3 mice model.
